# Delayed appendectomy versus early appendectomy in the treatment of acute appendicitis: a retrospective study

**DOI:** 10.1186/1749-7922-9-8

**Published:** 2014-01-21

**Authors:** Chang Sik Shin, Young Nam Roh, Jae Il Kim

**Affiliations:** 1 Department of Surgery, Ilsan Paik Hospital, Inje University College of Medicine, 170 Juhwaro, Ilsanseogu, Goyangsi, Gyeonggido, Republic of Korea

**Keywords:** Appendicitis, Early appendectomy, Delayed appendectomy

## Abstract

**Background:**

The controversy still exists about the timing of operation for appendicitis. The aim of this study was to compare the outcomes between early appendectomy and delayed appendectomy and assess the feasibility of delayed operation.

**Methods:**

The medical records of patients with acute appendicitis who received operation between January 1, 2011 and December 31, 2011, were retrospectively reviewed. Outcome measures were white blood cell (WBC) count at postoperative first day, time to soft diet, complication rate, surgical site infection (SSI) rate, length of hospital stay, and readmission within 30 days.

**Results:**

During the study period, a total of 478 patients underwent appendectomies, and 145 patients were excluded, leaving 333 who met inclusion criteria. Based on the time from arrival at hospital to incision, they were divided into two groups: 177 (53.2%) in group A and 156 (46.8%) in group B. There were no significant differences in preoperative demographics and clinical data between two groups. The mean WBC count at postoperative first day of group B were lower than that of group A (*p* = 0.0039). There were no significant differences in time to soft diet, length of postoperative hospital stay, complication rate, and readmission rate between two groups. SSI including intra-abdominal abscess was also shown no significant difference (Group A, 1.7% and Group B, 3.9%; *p* = 0.3143).

**Conclusions:**

This study revealed that delayed appendectomy was safe and feasible for adult patient although the clinical outcomes of delayed appendectomy were not superior to those of early appendectomy. We suggest that surgeons would decide the appropriate timing of appendectomy with consideration other situations such as available hospital resources.

## Introduction

Acute appendicitis has been the most common intra-abdominal condition requiring operation. Emergency appendectomy at the time of diagnosis was the standard of care for treatment of acute appendicitis during last century. Any delay in operation has been believed to increase postoperative morbidity or progress to complicated appendicitis such as perforated appendicitis or periappendiceal abscess [1,2].

However, the concept of emergency appendectomy has been recently challenged by studies which suggested that acute appendicitis could be treated medically, or delaying surgery did not show any increasing morbidity [3-7]. On the other hand, there are other studies which supported that appendicitis needed emergency surgical procedure and delay in surgery increased complication and length of hospital stay [8-10].

The controversy still exists about the timing of operation for appendicitis. The aim of this study was to compare the outcomes between early appendectomy and delayed appendectomy and assess the feasibility of delayed operation.

## Materials and methods

### Patients

This study was designed as a retrospective, observational study at a single institution. The medical records of patients with acute appendicitis who received operation between January 1, 2011 and December 31, 2011, were retrospectively reviewed. We excluded the following patients: (1) those who were under 16 years or over 65 years old, (2) those who underwent other surgical procedures along with appendectomy, such as cholecystectomy or oophorectomy, (3) pregnant women, and those with severe other medical disease requiring intensive care, (4) those who underwent incidental, interval, and negative appendectomies. The patients were then divided into two groups for comparison: Group A, those with a time from arrival to incision less than 8 hours and Group B, those with a time from arrival to incision longer than 8 hours.

### Data collection

The data were collected from the electronic medical records (EMR). The following parameters were included: demographics, duration from onset of symptoms to visit our hospital, time from arrival to diagnosis as appendicitis, time form diagnosis to operation, initial vital signs, initial laboratory findings, method of appendectomy, combined drainage procedures, pathologic findings, postoperative laboratory findings, time to a soft diet, postoperative complications, length of hospital stay, hospital costs, and readmissions within 30 days of surgery. We analyzed preoperative, operative, and postoperative clinical data obtained from each group. Hospital costs consisted of the total costs covered by National Health Insurance (NHI) and charges for non-covered items―non-covered bed charges or materials. The data on the following items were analyzed: the total hospital costs, the total costs covered by NHI and copayment by patients.

### Outcome measures

Outcome measures were white blood cell (WBC) count at postoperative first day, time to soft diet, complication rate, surgical site infection (SSI), length of hospital stay, and readmission within 30 days.

### Statistical analysis

The data were analyzed using SAS enterprise ver. 5.1 statistical software (SAS Inc, Cary, NC, USA). Demographics and clinical characteristics were expressed as means for continuous variables or proportions for categorical variables. The chi-square test was used to compare differences in categorical variables. Student’s *t* test or the Wilcoxon rank sum test was used to compare differences in continuous variables. The *p* value of less than 0.05 was considered statistically significant.

## Results

During the study period, a total of 478 patients underwent appendectomies, and 145 patients were excluded, leaving 333 who met inclusion criteria. Demographics and clinical characteristics of included cases are shown in Table 1. The mean age of patients was 35.4 years. There were 190 males (57.1%) and 143 females (42.9%). The average time from arrival at our hospital to diagnosis was 3.0 hours. The average time from diagnosis as appendicitis to skin incision was 6.6 hours. The average time form arrival to incision was 9.6 hours. Based on the time from arrival at our hospital to incision, they were divided into two groups: 177 (53.2%) in group A and 156 (46.8%) in group B.

**Table 1:** Demographics and clinical characteristics

**Total number of cases**	**333**
Age (years)	35.4 ± 12.4
Male: Female	190 (57.1%): 143 (42.9%)
Body mass index (kg/m^2^)	23.0 ± 3.3
Initial body temperature (°C)	37.4 ± 0.7
Initial white blood cell (WBC) count (×10^3^/mm^3^)	12.9 ± 3.9
Comorbidities	32 (9.6%)
Hours from onset of symptoms to hospital	24.3 ± 29.9
Hours from arrival to diagnosis	3.0 ± 2.0
Hours from diagnosis to incision	6.6 ± 4.7
Hours from arrival to incision	9.6 ± 5.0
Method of appendectomy (OA: LA)	248 (74.5%): 85 (25.5%)
Operation at night (22:00–06:00), case (%)	47 (14.1%)
Complicated appendicitis, case (%)	68 (20.4%)
Appendicoliths, case (%)	128 (38.4%)
Combined drainage, case (%)	63 (19.0%)
WBC, postoperative first day (×10^3^/mm^3^)	10.0 ± 3.3
Time to soft diet (day)	1.8 ± 1.0
Postoperative hospital stay (day)	4.6 ± 2.7
Complication, case (%)	11 (3.3%)
Readmission within 30 days, case (%)	2 (0.6%)

Comparisons of demographics and preoperative characteristics between two groups are shown in Table 2. There were significant differences in time parameter due to study design. There were no significant differences in age, sex ratio, body mass index (BMI), body temperature, initial WBC count, and comorbidities between two groups. Comparisons of operative characteristics between two groups are shown in Table 3. There were no significant differences in the ratio of laparoscopic appendectomy, operating time, the ratio of complicated appendicitis, and the ratio of accompanying external drainage procedure, and the ratio of accompanied by appendicoliths. There were significant differences between two groups in the ratio of operation at night (Group A, 22.0% and Group B, 5.1%; *p* < 0.0001), and in the ratio of accompanying external drainage procedure (Group A, 24.9% and Group B, 12.2%; *p* = 0.0033).

**Table 2:** Comparisons of demographics and preoperative characteristics between two groups

	**Group A (≤ 8 hours)**	**Group B (> 8 hours)**	***P *** **value**
Number of cases	177 (53.2%)	156 (46.8%)	
Age (yrs)	35.9 ± 125	34.7 ± 12.1	0.3758
Sex ratio (Male: Female)	103:74	87:69	0.6592
Body mass index (kg/m^2^)	23.1 ± 3.4	22.7 ± 3.1	0.2822
Body temperature (°C)	37.4 ± 0.7	37.4 ± 0.6	0.7701
Initial white blood cell count (×10^3^/mm^3^)	12.6 ± 3.8	13.3 ± 4.0	0.1150
Comorbidities	21 (11.9%)	11 (7.0%)	0.1915
Hours from onset of symptoms to hospital	26.4 ± 22.5	22.0 ± 16.7	0.1835
Hours from arrival to diagnosis	2.4 ± 1.1	3.6 ± 2.6	<0.0001
Hours from diagnosis to operation	3.4 ± 1.4	10.4 ± 4.3	<0.0001
Hours from arrival to operation	5.8 ± 1.5	13.9 ± 4.0	<0.0001

**Table 3:** Comparisons of operative characteristics between two groups

	**Group A (≤ 8 hours)**	**Group B (> 8 hours)**	***P *** **value**
Laparoscopic appendectomy, case (%)	42 (23.7%)	43 (27.6%)	0.4513
Operation at night (22:00–06:00), case (%)	39 (22.0%)	8 (5.1%)	<0.0001
Operating time (minute)	56.3 ± 21.8	53.5 ± 19.4	0.2236
Complicated appendicitis, case (%)	40 (22.6%)	28 (18.0%)	0.3408
Appendicoliths, case (%)	73 (41.2%)	55 (35.3%)	0.3097
Combined drainage, case (%)	44 (24.9%)	19 (12.2%)	0.0033

Comparisons of postoperative outcomes between two groups are shown in Table 4. The mean WBC count at postoperative first day of group B were lower than that of group A (p = 0.0039). There were no significant differences in time to soft diet, length of postoperative hospital stay, complication rate, and readmission rate between two groups. Although surgical site infection (SSI) rate including intra-abdominal abscess (IA) of group B was slightly higher than that of group A, there was also no significant statistical difference (Group A, 1.7% and Group B, 3.9%; *p* = 0.3143). Table 5 shows results of hospital costs between two groups and there were no significant differences in all comparative variables.

**Table 4:** Comparisons of postoperative outcomes between two groups

	**Group A (≤ 8 hours)**	**Group B (> 8 hours)**	***P *** **value**
WBC, postoperative first day (×10^3^/mm^3^)	10.5 ± 3.2	9.5 ± 3.3	0.0039
Time to soft diet (day)	1.9 ± 1.1	1.7 ± 0.8	0.0806
Postoperative hospital stay (day)	4.9 ± 2.8	4.4 ± 2.7	0.0719
Complication, case (%)	3 (1.7%)	8 (5.1%)	0.1225
Surgical site infection, case (%)	3 (1.7%)	6 (3.9%)	0.3143
Readmission within 30 days, case (%)	1 (0.6%)	1 (0.6%)	1.0000

**Table 5:** Comparisons of hospital costs between two groups

	**Group A (≤ 8hours)**	**Group B (> 8 hours)**	***P *** **value**
Total hospital costs	2,682,450 ± 733,183	2,618,006 ± 727,865	0.4225
Total costs covered by NHI	1,477,012 ± 378,827	1,449,149 ± 408,321	0.5189
Copayment by a patient	479,003 ± 115,575	461,984 ± 149,649	0.2511

Values are presented as KRW (Korean won, Korean monetary unit).

1 USD = 1,108 KRW.

NHI, National Health Insurance.

## Discussion

In Korea, the imaging modalities are so popular, and the payments are covered by national health insurance system. Radiologic evaluation could help surgeons to confirm the diagnosis and to recognize the location of appendix, and/or other intra-abdominal conditions requiring other procedures. All patients in this study received radiologic evaluation such as abdominal computed tomography (CT), abdominal ultrasonography and they were diagnosed with acute appendicitis.

Appendectomy has still been the most common non-elective surgical procedure performed by general surgeons [11,12]. It was usually prepared at the time of diagnosis as appendicitis and done within hours to prevent the progression of inflammation. However, the quality of antibiotics was improved in the last few decades and interval appendectomy for periappendiceal abscess was shown better outcomes than early operation. Recent studies suggested that periappendiceal abscess in selected cases could be managed by nonsurgical treatment without interval appendectomy [13,14]. Furthermore, successful results of nonsurgical antibiotics treatment for selected cases with uncomplicated appendicitis were reported in recent literatures [6,15,16]. However, at the present, we do not agree that appendicitis is medical disease.

Controversies regarding the timing of operation in patients needed operation still exist. Some studies still supported that the outcomes of immediate or prompt appendectomy were better than those of delayed appendectomy [8-10,17,18]. They advocated that delayed appendectomy produced more postoperative complication such as surgical site infection. On the other hand, some studies suggested that there was no significant difference of outcomes between early and delayed appendectomy [7,19,20]. In addition, several studies showed negative impact of prolonged working hours for residents or sleep deprivation on clinical performance and cognitive abilities [21,22].

The timing of surgery was actually affected by other factors such as limited operating room availability, limited anesthesia availability, limited equipment availability, as well as decision of a surgeon like results in survey of pediatric surgeons [23]. In our hospital, all of eight surgeons preferred early appendectomy and they performed appendectomy within a few hours after diagnosis except midnight, if possible. However, number of surgical residents was reduced and diseases to need operation were increased during last decade. Therefore waiting time to appendectomy has been naturally lengthened although early appendectomy was planned.

In our study, there were no significant differences in demographics, preoperative clinical characteristics between early appendectomy and delayed appendectomy groups. It means that disease severity such as fever, WBC count either uncomplicated or complicated appendicitis did not affect the timing of surgery. In addition, there was no significant difference in the ratio of accompanied by appendicoliths between two groups. In our study, the presence of appendicoliths did not affect the timing of surgery unlike with results of recent studies [24,25].

There were no significant differences in time to soft diet and length of postoperative hospital stay between two groups. There were also no significant differences in all parameters regarding hospital costs between two groups. Especially, there was no significant difference in complication rate including surgical site infection. One patient in group A and one patient in group B readmitted due to postoperative intra-abdominal abscess within 30 days. These results were similar with previous other studies [7,19,20]. Therefore delayed appendectomy is safe similar with early appendectomy.

Moreover, mean WBC count at postoperative first day of group B was lower than that of group A. These results might be due to sufficient and effective preoperative intravenous (IV) antibiotics injection to cover aerobic and anaerobic colonic flora [26]. In our hospital, when a patient was diagnosed as uncomplicated appendicitis by clinical and radiologic evaluation, IV cephalosporin (first or second generation) was given to the patient. If a patient was diagnosed as complicated appendicitis, IV metronidazole was added. As a result, patients in group A received single dose preoperative antibiotics and patients in group B received those twice or three times.

There are several limitations of this study. Firstly, this study was retrospective observational study. As above mentioned, several situations such as lack of resident, tight operation schedule made prospective study difficult. Secondly, optimal timing of appendectomy could not be elucidated. We expect to solve these limitations through the large prospective randomized trial in the near future.

## Conclusions

We still consider that appendicitis is not a medical disease but a surgical disease. This study revealed that delayed appendectomy was safe and feasible for adult patients with appendicitis although the clinical outcomes of delayed appendectomy were not superior to those of early appendectomy. Therefore, we suggest that surgeons would decide the appropriate timing of appendectomy with consideration other situations such as available hospital resources.

## Competing interests

This work was supported by the 2012 Inje University research grant. We declare that we have no competing interests.

## Authors’ contributions

CSS, YNR, and JIK carried out study design, acquisition and analysis of data, and drafted the manuscript. JIK carried out the statistical analysis. YNR and JIK revised the manuscript. All authors read and approved the final manuscript.
